# Pathophysiology and Management of Type 2 Diabetes Mellitus Bone Fragility

**DOI:** 10.1155/2020/7608964

**Published:** 2020-05-22

**Authors:** C. Eller-Vainicher, E. Cairoli, G. Grassi, F. Grassi, A. Catalano, D. Merlotti, A. Falchetti, A. Gaudio, I. Chiodini, L. Gennari

**Affiliations:** ^1^Unit of Endocrinology, Fondazione IRCCS Cà Granda-Ospedale Maggiore Policlinico, Milan, Italy; ^2^Istituto Auxologico Italiano, IRCCS, Unit for Bone Metabolism Diseases and Diabetes & Lab of Endocrine and Metabolic Research, Italy; ^3^Dept. of Clinical Sciences & Community Health, University of Milan, Milan, Italy; ^4^Ramses Lab, IRCCS Istituto Ortopedico Rizzoli, Bologna, Italy; ^5^Department of Clinical and Experimental Medicine, University of Messina, Messina, Italy; ^6^Department of Medicine, Surgery and Neurosciences, University of Siena, Italy; ^7^Department of Clinical and Experimental Medicine, University of Catania, University Hospital ‘G. Rodolico', Catania, Italy

## Abstract

Individuals with type 2 diabetes mellitus (T2DM) have an increased risk of bone fragility fractures compared to nondiabetic subjects. This increased fracture risk may occur despite normal or even increased values of bone mineral density (BMD), and poor bone quality is suggested to contribute to skeletal fragility in this population. These concepts explain why the only evaluation of BMD could not be considered an adequate tool for evaluating the risk of fracture in the individual T2DM patient. Unfortunately, nowadays, the bone quality could not be reliably evaluated in the routine clinical practice. On the other hand, getting further insight on the pathogenesis of T2DM-related bone fragility could consent to ameliorate both the detection of the patients at risk for fracture and their appropriate treatment. The pathophysiological mechanisms underlying the increased risk of fragility fractures in a T2DM population are complex. Indeed, in T2DM, bone health is negatively affected by several factors, such as inflammatory cytokines, muscle-derived hormones, incretins, hydrogen sulfide (H2S) production and cortisol secretion, peripheral activation, and sensitivity. All these factors may alter bone formation and resorption, collagen formation, and bone marrow adiposity, ultimately leading to reduced bone strength. Additional factors such as hypoglycemia and the consequent increased propensity for falls and the direct effects on bone and mineral metabolism of certain antidiabetic medications may contribute to the increased fracture risk in this population. The purpose of this review is to summarize the literature evidence that faces the pathophysiological mechanisms underlying bone fragility in T2DM patients.

## 1. Introduction

Osteoporosis and type 2 diabetes mellitus (T2DM) are chronic disorders associated with severe morbidity and increased mortality. Their prevalence, due to the general population ageing, is rapidly increasing and will early become a global epidemic imposing an overwhelming burden on health-care systems [1–7].

Nowadays, skeletal fragility is considered a complication of T2DM [1]. These patients have an up to 3-fold increased hip fracture risk [3–5]. Fractures of the wrist and the foot also seem to be more frequent, while the evidences on vertebral fractures are more limited [2]. Anyhow, available data suggest a higher risk of vertebral fractures and in particular morphometric vertebral fractures [6, 7], which has been suggested to be present in a third of T2DM postmenopausal women [8]. In T2DM patients, the fracture risk is increased for any given *T*-score with respect to the general population, so that fractures may occur despite a normal or even increased bone mineral density (BMD) [1, 5, 6], suggesting that the bone quality alterations rather than the BMD decrease may be the main factor influencing T2DM-related bone fragility [9]. Therefore, the assessment of BMD alone cannot represent a reliable tool to estimate fracture risk [9]. Similarly, fracture risk assessment algorithms, such as the WHO Fracture Risk Assessment Tool, underestimate fracture risk in these subjects [9–11].

Several pathophysiological mechanisms have been implicated in the deterioration of bone quality in T2DM. From a clinical point of view, the T2DM duration, the glycemic control, and the presence of the T2DM-related chronic complications (i.e., retinopathy, neuropathy, and macroangiopathy) are predictors of fragility fractures. Moreover, several T2DM therapies can have a direct negative role on bone metabolism. Chronic hyperglycemia and advanced glycation end product (AGE) accumulation, insulin resistance, altered bone marrow adiposity, inflammatory factors, and adipokines released by visceral fat and oxidative stress [2, 12] represent the principal mechanisms of T2DM-induced bone fragility.

Currently, the research in this field is getting rich by new evidences. Some data suggest that a decrease in hydrogen sulfide (H2S), which has a fundamental role for maintaining bone cell proliferation and differentiation, may be implicated in the pathogenesis of T2DM-related bone fragility [13]. Finally, starting from the similarities between the cortisol-related bone loss and T2DM-related bone fragility, the cortisol secretion, sensitivity, and peripheral activation (the so-called “cortisol milieu”) have been suggested to play a role in T2DM-related bone fragility [8, 14, 15].

This review is aimed at exploring the current understanding of the pathophysiological mechanisms underlying T2DM-related bone fragility.

## 2. Methodology

**Figure 1:**
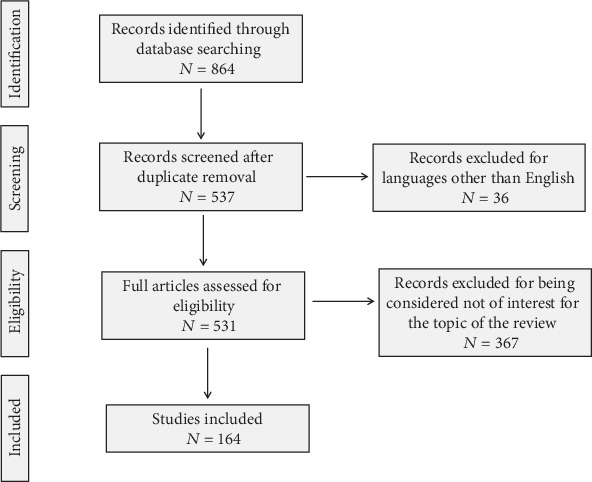
PRISMA flow diagram. According to PRISMA guidelines, PubMed and MEDLINE were searched from June 1968 to January 2020 for identifying published articles about bone metabolism and T2DM. In particular, we considered articles focused on the interactions between T2DM and bone fragility, such as hyperglycemia, insulin resistance, AGEs, bone marrow adiposity, inflammatory cytokines, H2S, and cortisol. Studies that analyzed how T2DM impacts on bone formation and resorption, collagen formation, skeletal muscle, and the incretin system were evaluated. Only publications in English were included.

According to PRISMA guidelines, PubMed and MEDLINE were searched from June 1968 to January 2020 for identifying published articles about bone metabolism and T2DM. In particular, we considered articles focused on the interactions between T2DM and bone fragility, such as hyperglycemia, insulin resistance, AGEs, bone marrow adiposity, inflammatory cytokines, H2S, and cortisol. Studies that analyzed how T2DM impacts on bone formation and resorption, collagen formation, skeletal muscle and the incretin system were evaluated. Only publications in English were included (Figure 1).

## 3. Evaluation of Bone Health in T2DM

### 3.1. Evaluation of Bone Fragility beyond Dual X-Ray Densitometry (DXA)

In T2DM, individual fractures generally occur at higher BMD levels than in nondiabetic subjects, with *T*-score levels being often above the osteoporotic range. Thus, it has been estimated that in T2DM subjects, an increase in hip fracture risk, similar than in controls, occurs at 0.4 and 0.6 SD higher BMD levels in men and women, respectively [3, 15–17]. On the other hand, the T2DM duration (i.e., >10 years), insulin treatment, and the presence of the T2DM-related chronic complications are associated with fragility fractures regardless of BMD. These evidences justify the need of a spinal X-ray in patients with T2DM chronic complications or poorly controlled disease, in addition to the already fractured ones. Indeed, up to a third of postmenopausal T2DM women investigated by a lateral spine radiograph showed asymptomatic morphometric vertebral fractures [8] that represent a major risk factor for additional subsequent fractures [18].

Again, considering the inadequate reliability of BMD in a T2DM population, other imaging techniques have been investigated in the last years [19]. Different retrospective and cross-sectional studies have showed that Trabecular Bone Score (TBS), a textural index based on evaluating pixel grey-level variations in the lumbar DXA image, providing an indirect index of bone architecture, is often reduced in T2DM [19] and that it might predict fracture risk better than BMD [15, 19–21].

In some cohorts of T2DM patients, the hip structural analysis (HSA) that represents an additional tool that can be applied to DXA in order to obtain information on bone geometry and indirectly assess the bone resistance to axial compressive forces [22] showed a weaker geometry (e.g., a narrower neck width) and compromised estimates of skeletal load response (e.g., a lower buckling ratio) [22]. However, the additive role of HSA on the prediction of fractures in T2DM remains to be established.

Although quantitative ultrasound (QUS) devices of the calcaneus and the phalanxes are widely available and low-cost techniques, therefore potentially very useful for the screening of large populations such as the diabetic one, limited information has been released about their use in T2DM. Available data showed that QUS parameters may be useful [23], but data concerning their predictive role in discriminating patients with and without fragility fractures are conflicting [23, 24]. Moreover, a correlation between reduced QUS parameters and poor glycemic control or peripheral nerve dysfunction has been also described [24].

Recently, peripheral quantitative computed tomography (QCT) and high-resolution peripheral QCT of the distal radius and tibia have been employed to obtain a 3D assessment of bone size, volumetric BMD, and bone macro- and microarchitecture (e.g., cortical porosity and trabecular connectivity). Moreover, QCT images can also be employed for the estimation of the mechanical properties of bone by means of finite element analysis (FEA) [19]. However, the study results using these techniques have been quite inconsistent. Several studies, although not all, suggest that in T2DM women, the indices of trabecular microarchitecture are preserved but cortical porosity is increased and it is specifically associated with a deficit in biomechanical properties, particularly in those diabetic females with fragility fractures [25–28]. Data in T2DM men are even more scarce, but available ones indicate that the deficits in cortical bone affect both sexes, at least in older T2DM patients [29].

The use of magnetic resonance imaging to assess trabecular and cortical bone parameters at both peripheral and axial skeleton could help in discriminating patients at higher fracture risk [25]. Remarkably, magnetic resonance spectroscopy of the vertebral bodies evidenced an altered bone marrow fat (BMF) composition (with lower unsaturation of bone marrow lipids) in T2DM postmenopausal women with fragility fractures [21]. This approach might represent a promising tool for fracture risk assessment in the future, given the negative role of BMF on bone health, as described later on [18, 25, 26].

Finally, an emerging invasive technique for direct measurement of mechanical characteristics of cortical bone in vivo is microindentation, which consists of the insertion of a specific probe into a cortical bone's surface at the anterior tibia to induce microscopic fractures. The impact microindentation, from which a ratio called bone material strength index (BMSi) can be derived as an index of fracture resistance, has been used for the assessment of bone properties in T2DM. Postmenopausal T2DM women showed significantly lower BMSi compared to controls, also after adjustment for BMI and despite similar BMD. Moreover, BMSi values were found to be negatively correlated with glycemic control and disease duration [19], thus confirming, using a direct in vivo measure, compromised bone quality in T2DM and the potential detrimental effects of prolonged hyperglycemia on bone.

Currently, the other available techniques beside DXA and vertebral morphometry, notwithstanding their potential role for investigating the mechanisms of the T2DM-related bone fragility, need to be tested in prospective studies and their scarce availability, high costs, microindentation and also the invasive nature of the procedure do not consent a routine use in clinical practice.

In addition to the indications in nondiabetic patients, a spinal radiograph for evidencing possible asymptomatic vertebral fractures should be performed in T2DM patients with clinical fragility fracture and/or with T2DM-related chronic complications, insulin use, and/or long T2DM duration (i.e., above 10 years).

### 3.2. Bone Turnover

In T2DM patients, histomorphometric studies have shown a reduction of the osteoblast number and of the osteoid amount [30] and a low bone formation rate [31]. Interestingly, this reduction in bone formation and mineralized surface has been found in the cancellous, intracortical, and endocortical surfaces of patients with T2DM but not of patients with type 1 diabetes [32]. However, some other data suggested that in addition to the reduction of the activation frequency of the bone remodeling units, in some patients, an increase in bone mineralization may be present. However, in T2DM, the nonenzymatic collagen crosslinking by pentosidine was found to be increased and directly associated with glycated hemoglobin (HbA1c) levels [33]. Overall, these data suggest a low bone turnover state in T2DM. Interestingly, the pentosidine level has been proposed as a bone fragility marker in T2DM [34, 35].

In keeping with these data, even clinical studies have shown a reduced bone turnover in T2DM. In particular, both bone apposition, as mirrored by osteocalcin levels, and bone resorption, as evaluated by the C-terminal telopeptide of type I collagen (CTX) levels, were found to be reduced and negatively associated with metabolic control [36–38]. In keeping with a reduction of bone turnover in T2DM, other markers of bone apposition and resorption, such as the procollagen type 1 amino-terminal propeptide and the N-terminal telopeptide of type I collagen, respectively, were found to be reduced in patients with T2DM than in nondiabetic controls [39]. At variance, alkaline phosphatase total activity has been found to be increased in T2DM patients than in nondiabetic individuals. Even though both histomorphometrical and biochemical data point toward a low bone turnover osteoporosis, some data seem conflicting [37]. It must be considered, however, that the different studies are frequently not easily comparable, due to differences in disease duration, glycometabolic control, presence of chronic complications, age, ethnicity, and several other differences among study participants. Importantly, notwithstanding the potential role of a decrease of bone turnover in reducing the microcrack repairing in T2DM patients and, thus, in increasing the fragility fracture risk, we still do not know whether bone turnover markers can be used to predict fractures in T2DM patients.

Even a role of low levels of parathyroid hormone (PTH) has been hypothesized in T2DM-related bone fragility [40]. Indeed, some data suggested that a subtle hypoparathyroidism could contribute to low bone turnover in patients with diabetes mellitus. In keeping with this idea, PTH levels have been found to be directly associated with CTX, tartrate-resistant acid phosphatase 5b, and osteocalcin levels [40]. Interestingly, a chronic hypomagnesemia has been hypothesized to impaired PTH secretion in T2DM [41], and a renal calcium leak induced by glycosuria can determine a negative calcium balance, which seems to normalize after improving the glycometabolic control [42].

Summarizing, in the authors' opinion, the use of bone turnover markers and/or PTH level determination are not to be considered mandatory in the vast majority of T2DM patients. The determination of CTX and PTH levels should be reserved in doubtful cases (for example, if an additional secondary cause of osteoporosis is suspected) and on case-by-case basis.

## 4. Factors Leading to Bone Fragility in T2DM

### 4.1. Disease Duration, Insulin Use, Glycometabolic Control, and Complications

The difficulty in discriminating the independent effects of disease duration, metabolic control, and presence of T2DM complications is due to the existence of a strong link between these aspects of the T2DM condition. However, different studies found that a T2DM duration longer than 10 years significantly increases the fragility fracture risk, regardless of diabetes control [43–46]. Generally, the T2DM duration seems to negatively affect bone metabolism, even though it is important to keep in mind that T2DM may often remain undiagnosed for many years. Besides T2DM duration, a poor glycemic control (e.g., HbA1c levels ≥ 7.5%) has been shown to be associated with increased fracture risk [47, 48]. A large (i.e., enrolling more than 4 thousand individuals) long-term prospective (i.e., about 12 years mean follow-up) study showed that the fracture risk is similar between nondiabetic subjects and adequately controlled T2DM patients [49], while the fracture risk was 1.6-fold increased in subjects with inadequately controlled T2DM. This relationship between T2DM control and fragility fracture risk was not confirmed in another study, in which, however, the median levels of HbA1c were only slightly elevated (~7.5%) [50]. Despite these evidences, it is important to keep in mind that the predictive value of a single HbA1c value in the determination of fracture risk is questionable.

Whether T2DM complications could represent independent risk factors for bone fragility is still a matter of debate. In a large case-control study, T2DM itself and all its complications were significantly associated with an increased overall risk of fractures [43, 51, 52], without a clear evidence of the independent contribution of each single factor. Interestingly, both neuropathy and insulin use may influence the risk of falls [53–55], which are of crucial importance in these patients, being associated with an increased risk of fracture, hospitalization, and death [56]. Indeed, as compared to nondiabetic subjects, the risk of falling more than once a year is known to be increased in older women with T2DM without insulin use and even higher in insulin users [54]. Sarcopenia, the age-related decline in skeletal muscle mass, quality, and function, may represent an additional contributing factor to the increased fall and fracture [57], and it is known to be associated with T2DM. Indeed, in T2DM patients, both muscle strength and function are decreased as compared to nondiabetic subjects [58, 59]. In addition, T2DM patients can present neuromuscular dysfunction, which may favor falls irrespective of sarcopenia [60]. However, the evidences of association between sarcopenia, fall risk, and bone fragility in T2DM cohorts are still limited.

Finally, some studies found an association between microvascular disease and bone microstructure as well as with fracture risk. This association might be explained by an altered vascular supply to the skeleton, in particular cortical bone, that may contribute in compromising bone formation [2].

Overall, in our opinion, great attention should be reserved to T2DM patients, who have a long-standing disease and/or chronic complications and/or are insulin treated.

### 4.2. Oral Antidiabetic Agents

The effect of oral antidiabetic agents on bone is summarized in Table 1. Among the possible mechanisms contributing to the increased risk of fracture in T2DM, the use of antidiabetic drugs with direct effects on bone cells or an indirect effect on bone metabolism has to be taken into consideration.

Metformin, the worldwide accepted first-line drug in the treatment of T2DM [61], exerts its effect decreasing liver glucose production, enhancing insulin sensitivity, and inhibiting fatty acid synthesis and promoting their oxidation [62]. In most animal studies, metformin seems to improve bone mass and strength [2, 63], by preventing the advanced glycation end product (AGE) accumulation, known to induce alterations in the osteoblastic cells. Moreover, metformin has been suggested to inhibit the formation of reactive oxygen species (ROS) and apoptosis in osteoblastic cultures exposed to high glucose concentrations [63]. Unfortunately, the evidence of a positive effect on bone of metformin administration in T2DM patients is less impressive and somewhat conflicting. However, overall metformin is reported having positive or neutral effects on fracture risk in T2DM patients [46, 64–66].

The role of sulfonylureas (insulin secretagogues, blocking ATP-regulated K+ channels, that enhance insulin release from pancreatic *β*-cells) on bone metabolism has been investigated only in few studies [61]. Available data evidence a potential stimulatory effect on osteoblast proliferation and differentiation and a protective role on osteoblasts against hyperglycemia [2, 63]. However, some studies reported an increased risk of falls and fractures that might be due to the increased risk of hypoglycemia associated with the use of these drugs [64, 67], while other studies reported a neutral or even positive effect on fracture risk [46, 65–69].

Pioglitazone and rosiglitazone, known as thiazolidinediones (TZDs), activating peroxisome proliferator-activated receptor gamma (PPAR*γ*), reduce the extent of insulin resistance and improve *β*-cell response towards altered glucose levels. Despite the beneficial effect of TZDs on glycemic control, their prolonged use has been associated with negative effects on bone metabolism. Interestingly, in bone marrow stromal stem cells (BMSC), PPAR*γ* activation increases adipogenesis and decreases osteoblastogenesis [70–72]. In keeping, TZDs have been shown to decrease bone formation, increase osteoclastogenesis, and promote osteocyte apoptosis [2, 63]. Several clinical studies have shown that in patients using TZDs, the bone formation markers decrease, while the bone resorption markers increase and BMD declines [2, 63]. Moreover, randomized controlled trials and prospective studies revealed an increased peripheral fracture risk in TZD-treated patients, especially in postmenopausal T2DM women [72–76]. Furthermore, BMD loss observed in TZD users seems to be not reversible after treatment discontinuation [77].

Gastric inhibitory polypeptide (GIP) and glucagon-like peptide 1 (GLP-1) are gut-derived hormones that stimulate insulin, suppress glucagon secretion, inhibit gastric emptying, and reduce appetite and food intake (so-called “incretin effect”). Patients with T2DM have a reduced incretin effect [78]. The therapeutic approaches for restoring the incretin action include degradation-resistant GLP-1 receptor agonists (GLP-1 mimetics) and inhibitors of dipeptidyl peptidase-4 (DPP-4) activity [79]. The presence of GLP-1 and GIP receptors in human osteoblastic cells at different stages of differentiation induced many authors to investigate the effect of these gut-derived hormones on bone metabolism [80]. Moreover, GLP-1 receptors are expressed even in BMSC and immature osteoblasts [81]. Several evidences suggest that GLP-1 stimulates proliferation of mesenchymal stem cells and inhibit their differentiation into adipocytes [82]. In vivo studies showed an osteogenic effect of GLP-1 that seems to be mediated through the inhibition of the expression of the sclerostin gene [83] and of the WNT pathway [81]. A study in rodents showed that the higher the doses of exendin-4 (a GLP-1 mimetic), the higher the increase in bone strength and bone formation [84].

From a clinical point of view, few meta-analyses or post hoc analyses of population-based studies have been performed on the relation between the incretin use and bone fragility in T2DM and showed conflicting results. A recent meta-analysis of 16 RCTs on the effect on fracture risk of the GLP-1 receptor agonists showed that, among the GLP-1 mimetics, while the exenatide use was associated with an increased fracture risk with respect to other antidiabetic agents, the liraglutide use was associated with a significantly reduced risk of fractures [85]. However, other studies did not report significant effects of GLP-1 receptor agonist fracture risk and BMD [86, 87]. Also, for DPP-4 inhibitors, available data are conflicting. In vitro studies show a neutral effect on osteoblast differentiation. However, in animal models, these agents have been found to increase trabecular and cortical bone volume, due to a suppression of bone resorption [2, 63]. As far as study in humans is concerned, although two clinical studies showed a positive effects on fracture prevention in patients treated with the DPP-4 inhibitors [88, 89], a recent meta-analysis reported a neutral role of these agents [90]. Overall, it should be underlined that none of these studies were specifically designed to assess the effect of DPP-4 inhibitors or of incretins on fracture prevention, and the information regarding fractures has been obtained only by analyzing the safety profile. This explains the small fracture number emerged from these studies.

A new class of blood glucose-lowering drug for T2DM is represented by the sodium-glucose cotransporter-2 (SGLT-2) inhibitors. These drugs inhibit SGLT2 in the proximal convoluted tubule preventing the reabsorption of glucose and inducing its excretion in urine. Importantly, even the tubular phosphate reabsorption is increased by using these agents. Available preclinical and clinical data suggest that the SGLT2 inhibitors might negatively affect bone health, but data on fracture risk are controversial [2]. Indeed, two pooled analyses of RCTs reported neutral effects of SGLT-2 inhibitors on fracture [91–93], while other studies found an increased fracture incidence, more evident with the use of canagliflozin, with fractures occurring already after 12 weeks of drug initiation and increasing over time [94–96]. At present, it is not clear whether the bone negative effects of SGLT-2 inhibitors are mechanism-based or compound-specific.

Even though no specific study regarding oral antidiabetic agents and fracture risk is available, in our opinion, a particular attention at bone health should be paid in patients treated with TZDs and/or canagliflozin.

### 4.3. Insulin

The available data on the effect of insulin on bone are summarized in Table 1. In the presence of a treatment failure with the oral antidiabetic medications, insulin therapy represents the elective therapy for T2DM patients. In preclinical studies, insulin seems to play an important role in bone metabolism, in keeping with the presence of insulin growth factor receptors (IGFRs) on the surface of both osteoclasts and osteoblasts. *In vivo* and *in vitro* studies established that insulin exerts an anabolic effect on bone [97]. Mice with altered insulin signaling, due to the lack of IGFRs, have low bone turnover and reduced BMD [98]. On the other hand, insulin injection is able to induce bone formation, inhibit bone resorption, and lead to BMD improvement in adult mice [99].

At variance, in most clinical studies, the positive effect of the insulin treatment on both bone turnover markers and BMD [100] is not evident. Rather, its use has been associated with a higher risk of fractures (in particular, nonvertebral ones) [43, 46, 64, 101]. In a recent study on a large cohort of T2DM patients, insulin monotherapy was clearly associated with a 1.6-fold increased fracture risk in respect with metformin monotherapy [102]. However, recent data show that the use of long-acting insulins, less apt to induce hypoglycemia, was associated with a lower fracture risk as compared to other insulins [103], suggesting that, at least in part, the higher fracture risk associated with the insulin use might depend on a higher risk of hypoglycemia-related fall. Overall, it should be considered that insulin-treated T2DM patients have generally a longer disease duration and a higher number of comorbidities that could *per se* influence the fracture risk, regardless of the insulin use.

Eventually, is important to note that, although a relative insulin deficiency occurs in the later stages of T2DM, the predominant defect in this condition is the insulin resistance. We still do not know how insulin resistance affects bone and whether or not the skeletal loading might be compromised due to decreased muscle strength secondary to decreased glucose uptake by muscles.

As already mentioned, in the authors' opinion, insulin treatment has to be considered a risk factor for fragility fracture in T2DM patients.

### 4.4. Glucose Toxicity

**Figure 2:**
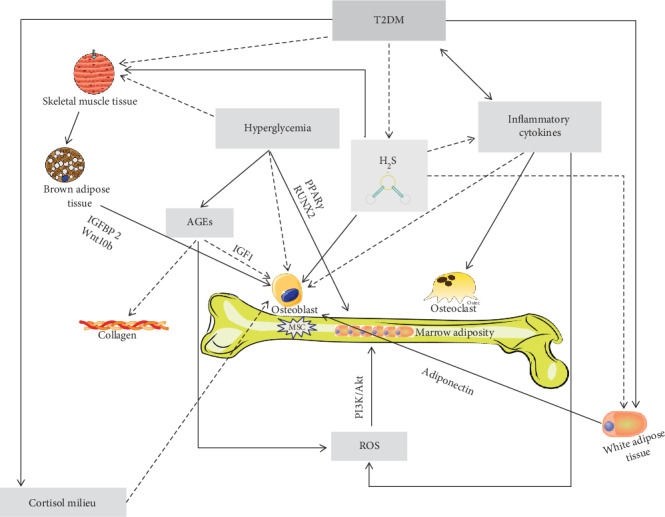
Mechanisms underlying bone fragility in type 2 diabetes mellitus (T2DM). In T2DM, the muscle tissue reduction, due to several factors including hyperglycemia per se, but probably also hydrogen sulfide (H2S) decrease, is thought to have a negative role on osteoblast lineage, via its crosstalk with the brown adipose tissue. Indeed, the muscle tissue is known to influence the brown adipose tissue, physiologically stimulating the secretion of factors (such as IGFBP2 and Wnt10b) thought to be important for osteoblast proliferation and activity. Osteoblast differentiation and activity, in T2DM, may be also impaired directly by the reduction of H2S levels that physiologically are thought to stimulate the osteoblast lineage. Hyperglycemia may directly reduce bone mesenchymal stem cell (MSC) viability and clonogenicity and also have an indirect negative effect on osteoblasts via the accumulation of advanced glycation end products (AGEs), which negatively affects osteoblasts through a reduction of the insulin-like growth factor-1 (IGF1) levels. The AGE accumulation impairs the normal collagen formation and leads to reactive oxygen species (ROS) increase that may augment marrow adiposity via the phosphoinositide-3-kinase–protein kinase B/Akt (PI3K/Akt) pathway. The inflammatory cytokine increase, directly and/or indirectly (due to the H2S reduction), may also impair osteoblastogenesis and increase osteoclast activity and ROS, ultimately leading to bone adiposity. Finally, in T2DM, osteoblasts may be also damaged by the low adiponectin levels due to the increase of white adipose tissue, which is a characteristic of T2DM itself but also a consequence of low H2S levels. Finally, even an altered cortisol secretion, peripheral activation, and sensitivity (i.e., “cortisol milieu”) have been suggested to potentially impair osteoblast activity.

As evidenced above, many evidences point toward a reduced bone turnover in T2DM, with a negative correlation between glycometabolic control and bone apposition and resorption markers. Hyperglycemia exerts troublesome effects on osteoblastogenesis since the early steps of differentiation, ultimately leading to low bone turnover. High blood glucose levels may reduce MSC viability and clonogenicity [104]. Several in vitro studies showed, in the presence of hyperglycemia, a downregulation of the BMSC proliferation, osteoblast gene expression, alkaline phosphatase (ALP) activity [105], and bone mineralization rate in BMSC isolated from streptozotocin- (STZ-) induced diabetic rats [105]. In addition, BMSC exposed to chronic high glucose exhibit enhanced adipogenic rather than osteogenic pathway, due to the PPAR*γ* activation, and an enhanced expression of cyclin D3 [106] and decreased Runt-related transcription factor 2 (RUNX2) [107], ALP [108], and osteocalcin expression in osteoblasts. In keeping, studies in animal models confirmed a reduced mineralization and decreased trabecular bone volume in T2DM, probably due to the decreased RUNX2 gene expression and to reduction of osteocalcin, osteoprotegerin, bone morphogenetic protein-2 expression, and ALP [109–113] (Figure 2).

Recently, some evidences suggest that even the osteocytes, the most abundant bone cell type orchestrating bone remodeling, are affected by hyperglycemia. Indeed, in T2DM, sclerostin and Dickkopf-related protein 1 (Dkk1), two major contributors of bone formation via Wnt signaling inhibition, are increased and *β*-catenin is reduced [109]. Increased serum levels of sclerostin have been observed in T2DM patients [114] and have been shown to be associated with vertebral fractures [115]. In addition, in T2DM patients, the usual PTH-induced transcriptional suppression of sclerostin production is lost. In keeping, the treatment with sclerostin antibodies improves bone mass and strength in T2DM animal models.

Overall, these cellular and animal models indicate that in T2DM, a preferential differentiation of the BMSC toward adipocytes rather than osteoblast lineage is present. Interestingly, even clinical data are in line with this theory. Indeed, recent studies show that in T2DM, an inverse association exists between marrow adipose tissue (MAT) and glycemic control and T2DM women with poor glycemic control have significantly higher MAT levels than those with adequate glycemic control [116, 117]. The functional significance of MAT and its implications for bone quality remain to be clarified, as well as the relationship between MAT and other fat depots (i.e., visceral and subcutaneous fat stores) and possible hormonal determinants. Interestingly, another possible mechanism that may elucidate the prevalence of adipogenesis on osteoblastogenesis is the PI3K/Akt pathway, which is activated by the reactive oxygen species (ROS) production, which, in turn, is associated with hyperglycemia (Figure 2).

We know that AGE levels are increased in T2DM as a result of prolonged hyperglycemia and oxidative stress. The activation of AGE receptor, expressed in human-derived bone cells, enhances inflammatory cytokine production and ROS production, triggering a vicious cycle of inflammation and bone resorption [118]. Moreover, AGEs may reduce the expression of RUNX2, osteocalcin, and osterix [119], which are well-known important factors in osteoblast differentiation. Furthermore, on the one hand, AGEs suppress endoplasmic reticulum function, essential to osteoblast differentiation and activity [120] and, on the other hand, they increase osteoblast apoptotic death [119]. All these mechanisms induce a reduction of mineralization [31, 121] and a bone quality impairment. Finally, hyperglycemia may play a negative role also in osteoclastogenesis, inducing an impaired bone resorption. Hyperglycemia could especially impair embryonic stem cell differentiation in osteoclast, usually promoted by physiological glucose levels. In keeping with this, STZ-induced diabetic mice present impaired bone resorption due to reduced levels of dendritic cell-specific transmembrane proteins involved in osteoclast differentiation [122, 123]. However, other evidences show elevated bone resorption and osteoclast activity [111, 113] that may compromise the mineralization [124] (Figure 2).

Hyperglycemia exerts its negative effect on bone health also acting on extracellular matrix. It is well known that bone elasticity, toughness, and strength are dependent on the type of cross-links between the adjacent collagen molecules, while the mineral component of the bone matrix provides stiffness. Indeed, while enzymatic cross-links are essential to maintain bone strength, the formation of nonenzymatic AGE cross-links within collagen fibers negatively affects bone strength [125]. The low turnover of collagen leads to the accumulation of a huge quantity of altered type 1 collagen, which may induce, at both trabecular and cortical levels, biomechanical changes [126]. In addition, both in vitro and in vivo animal and human studies demonstrated that trabecular bone is susceptible to the accumulation of nonenzymatic glycation, which increases its propensity to fracture and decreased flexion strain and energy (Figure 2).

In an animal model, pentosidine levels and the pentosidine/total enzymatic cross-link ratio were negatively associated with some mechanical bone properties such as energy absorption, stiffness, maximum load, and elastic modules [127]. Plasma and/or urinary pentosidine has been investigated as a potential clinical marker of bone damage in T2DM. In a T2DM Japanese cohort, pentosidine levels have been found significantly higher in postmenopausal women with vertebral fractures [34] than in nonfractured ones, regardless of the glycemic control, BMD, other osteoporosis risk factors, and renal function, all factors known to affect pentosidine levels.

Although these observations suggest that the impairment in collagen cross-links and AGE formation might explain the reduced bone quality in T2DM, larger and more robust studies are needed to confirm this hypothesis and to allow pentosidine being used as a marker for fracture prediction in T2DM patients.

### 4.5. Insulin Growth Factor 1 (IGF1), Inflammatory Cytokines, Brown/Beige Fat, and Adipokines

In T2DM, bone fragility is conceivably linked to an altered regulation of insulin growth factors (IGFs). Several *in vivo* studies have shown that high concentration of AGEs blunt the stimulatory IGF1 action on osteoblasts, probably through an osteoblast resistance to the IGF1 action [128, 129]. In postmenopausal women affected with T2DM, IGF1 were found to be inversely associated with the presence and the number of vertebral fractures, regardless of T2DM control, age, spinal BMD, renal function, and insulin secretion [130] (Figure 2).

Overall, T2DM is often described as a state of accelerated ageing. Inflammatory cytokines have been embroiled in the T2DM development as well as in its micro- and macrovascular complications. Inflammatory cytokines seem to have a role also in T2DM-related bone disease. Indeed, osteoclastogenesis can be activated by elevated cytokine levels, while osteoblast differentiation can be suppressed [131, 132]. Importantly, obese T2DM subjects show significantly higher levels of interleukin-6 and tumor necrosis factor-alfa that, at tissue level, may induce the ROS production, therefore affecting differentiation and survival of osteoblasts, osteoclasts, and osteocytes [133] (Figure 2).

Brown adipose tissue, which is typically thermogenically active, has been found to be reduced in T2DM and obesity [134]. Insulin-like growth factor-binding protein-2 and Wnt10b, factors secreted by brown adipose tissue, have an anabolic effect on bone metabolism and increase osteoblast activity [135]. In addition, the inactivation of TGF*β*–SMAD3–myostatin signaling [136] promotes the browning of adipocytes. These recent data encourage the development of a novel class of TGF*β*–myostatin antagonists that could be potentially used to treat both obesity and the T2DM-related bone disease (Figure 2).

Dysregulation of serum adipokine levels is also possibly linked to the T2DM-related low bone turnover. Indeed, T2DM patients present low adiponectin levels, an adipokine exclusively produced by the adipose tissue [137]. In vitro, adiponectin seems to have an inhibitory effect on osteoclasts and an anabolic effect on osteoblasts [138]. However, the studies aimed at investigating the link between adiponectin and BMD gave conflicting results, some data showing an inverse relationship [139, 140], while others showing a positive relationship between adiponectin and BMD at distal radius [141]. Furthermore, T2DM patients present low levels of leptin, another adipokine produced by white adipose tissue as well as by osteoblasts and bone marrow adipocytes. A Japanese study showed a significant negative correlation between leptin and bone resorption in T2DM subjects. Moreover, these authors showed that distal radius BMD was associated with leptin levels, but this association was not present for spine and hip BMD [141]. These results suggest that adipokines may exert a differential effect on cortical versus trabecular bone [142].

Further research is needed to confirm if the adipokine levels may be associated with bone disease in T2DM and if their determination may be useful in the clinical practice (Figure 2).

### 4.6. Obesity, T2DM, and Bone Fragility: A Concept of “Circular Health” in Body Energy Control

Human health can be regarded as a system of communicating vessels, particularly true when abnormalities in the management of the energy balance exist. The concept of “circular health” would suggest an interdisciplinary approach to identify and treat the multifactorial determinants of chronic diseases. The ability to adapt and adjust to different environmental conditions has been enabling the humans in survival. In a period of famine or hunger, the following conditions occur: (a) decrease in basal metabolic rate, leptin production, muscle mass, and lipolysis and (b) increase in cortisol secretion and lipogenesis. The opposite occurs when abundant food is available. Currently, in industrialized countries, rarely radical fluctuations in diet and metabolism occur, and, consequently, unfavorable health conditions such as obesity and diabetes mellitus develop. Over the past 20 years, it has been suggested that human skeleton may exert an important role also in energy metabolism through “local” hormonal connection, such as adipokines, mainly released by adipose tissue, but not exclusively, insulin/insulin-like growth factor 1 (IGF1), and osteocalcin/undercarboxylated osteocalcin pathways [143, 144], together with organs known to be involved in metabolic control. Such molecular pathways may be fundamental in maintaining energy homeostasis by controlling and coordinating both “fuel” uptake and energy expenditure in the human body, probably within a more complex network in which also central nervous system neurons and peripheral energy centers, sensing and regulating the energy needs, cooperate. The common cellular origin of osteoblasts, myocytes, and adipocytes makes not surprising the hypothesis that the skeleton either has a role in energy metabolism or may suffer in both skeletal muscle and adipose cell diseases, even if the underlying molecular mechanisms involved are still to be understood. Diabetic animal models and in vivo human studies suggested a strict interaction between whole body metabolism and skeletal health. It is well known that obesity and T2DM have a negative impact on fracture risk, but the knowledge on possible interactions of obesity, T2DM, and fracture still needs to be elucidated. Several studies suggested that alterations of adipose tissue-released hormones, such as adipokines, may exert harmful effects on bone cells. In particular, an in vitro study revealed that adiponectin, produced by adipose tissue, may exert either an anabolic effect on osteoblasts or an inhibitory effect on osteoclasts [138], and low levels of adiponectin are found in patients with T2DM [137]. However, conflicting results concerning a clinical evidence on the link between adiponectin and BMD exist [139–141]. Impaired leptin production, produced by white adipose tissue, bone marrow adipocytes, and osteoblasts, has been observed in diabetic patients, and a significant negative correlation between its serum levels and the bone resorption marker urinary NTX has been reported in T2DM Japanese subjects, who showed a significant positive correlation between serum levels of leptin and *Z*-scores at the distal radius but neither at the lumbar spine nor at femoral neck levels, as if a differential effect on cancellous versus cortical bone existed [141, 142]. A fracture-related morbidity seems to be a higher in obese than in nonobese women [145]. Higher fat depots negatively act on bone, and the cytokines produced by visceral fat exhibit a proresorptive effect while an increased intramuscular fat accumulation associates with a reduced and less effective skeletal muscle function, powering both the attenuation of loading effects and the increase of risk for falls typically observed also in T2DM [146]. Metabolic syndrome, a cluster of cooccurring conditions highly increasing the risk for cardiovascular heart diseases, T2DM, excess body fat around the waist, and abnormal cholesterol or triglyceride levels, and dysmobility syndrome, a cluster of coexisting conditions such as osteoporosis, sarcopenia, obesity, ultimately increasing the risk for falls and fractures in affected subjects [147], may coexist in obese-T2DM patients. However, a common denominator in both syndromes, represented by higher individual fragility and impairment of the energy balance of the body, either as its generation or its dissipation/transformation, can be hypothesized. The importance of these metabolic pathways is underlined by common metabolic diseases, such as osteoporosis, diabetes, and obesity, caused by genetic or environmental disturbances in endocrine control mechanisms. The impact of coexisting obesity and diabetes determines rising health costs and disability, other than a poor health status.

In a circular health model, a common multitasking diagnostic-clinical-therapeutic management of these patients is to be recommended [148, 149].

### 4.7. Cortisol Secretion, Peripheral Activation, and Sensitivity

**Figure 3:**
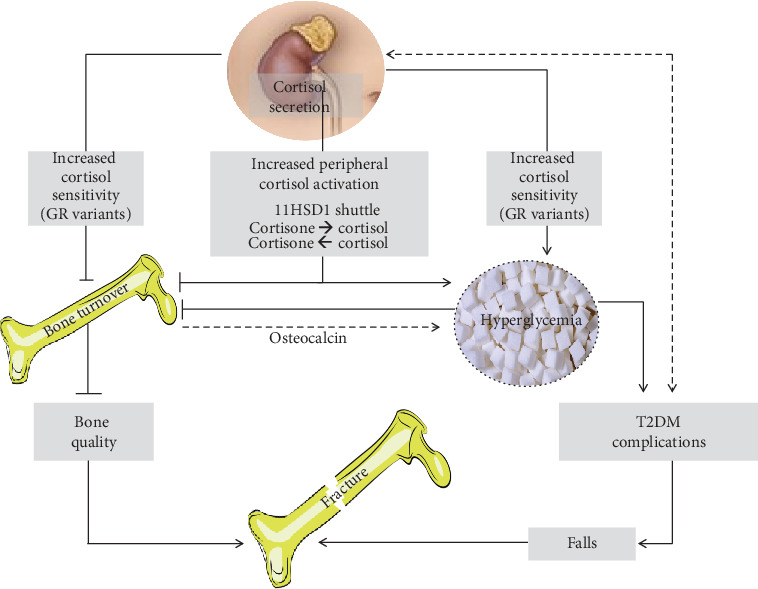
Cortisol milieu and bone fragility in type 2 diabetes mellitus (T2DM). In T2DM patients, an increased (even though still within the normal range) cortisol secretion is present, particularly in those affected with the diabetic complications, which in turn is hypothesized to be a trigger for the increased cortisol secretion itself. The sensitizing variants of the glucocorticoid receptor (GR) may increase the negative effect of cortisol on both T2DM control and bone metabolism, contributing to the shift in the balance between osteoblastogenesis and adipogenesis of mesenchymal stem cells in bone. The degree of the interconversion of cortisone in cortisol, due to the activity of the 11*β*hydroxysteroidodehydrogenase type 1 (11HSD1), may influence bone metabolism in T2DM. Indeed, in humans, the selective inhibition of 11HSD1, which has been even suggested as potential treatment for T2DM, has been also demonstrated to improve diabesity and osteoblast differentiation in a mouse model. Finally, in T2DM, a vicious circle could be hypothesized between the increased cortisol secretion, peripheral activation, and sensitivity (i.e., “cortisol milieu”) and bone and glycometabolic control. Indeed, the low bone turnover induced by this activated cortisol milieu could contribute in reducing the undercarboxylated osteocalcin levels, which decrease and, in turn, may worsen the glycometabolic control, therefore perpetuating the mechanisms leading to reduced bone turnover. The final effects of these alterations of the cortisol milieu in T2DM may be on one side of the reduction of bone quality, since the low bone turnover reduces the possibility of the microcrack repairing, and, on the other side, the worsening of the T2DM complications that ultimately could lead to an increased risk of falls. The reduction of bone quality together with the increased risk of falls is among the most important factors associated with bone fragility in T2DM.

The low bone turnover with a decreased osteoblastic function typical of the T2DM-related bone damage is also a feature of the glucocorticoid-induced osteoporosis. Interestingly, in T2DM patients, the cortisol secretion and/or sensitivity have been suggested to influence the diabetic disease. Indeed, in T2DM patients, an increased (even though still within the normal range) cortisol secretion is present, particularly in those affected with the diabetic complications [13] and the different glucocorticoid receptor (GR) gene polymorphisms have been found to potentially influence the disease control [150]. Interestingly, the cortisol secretion and sensitivity (as represented by the N3S3S sensitizing variant of GR gene) have been suggested to be associated with the presence of asymptomatic vertebral fractures in postmenopausal T2DM patients [8, 14]. These clinical data, suggesting a potential role of the degree of cortisol secretion and sensitivity in the T2DM-related bone osteoporosis, are in line with recent in vitro data showing that the shift in the balance between osteoblastogenesis and adipogenesis of MSC may be mediated by the GR genetic variants [151]. In addition, even the degree of the interconversion of cortisone in cortisol at the peripheral tissue levels (including bone), due to the activity of the 11*β*hydroxysteroidodehydrogenase type 1 (11HSD1), may influence bone in T2DM. Indeed, the selective inhibition of 11HSD1, which has been suggested as potential treatment for T2DM in humans [152], has been also demonstrated to improve diabesity and osteoblast differentiation in a mouse model [153]. Finally, in T2DM, a vicious circle could be hypothesized between cortisol “milieu,” bone, and glycometabolic control. Indeed, the low bone turnover induced by the increased cortisol secretion, peripheral activation, and sensitivity could contribute in reducing the undercarboxylated osteocalcin levels [154], which, in turn, could worsen the glycometabolic control, eventually leading to a perpetuation of low bone turnover (Figure 3).

These data may have an important clinical application. Indeed, if bone damage in T2D were related, at least partially, to the degree cortisol secretion and/or sensitivity, the treatment with an 11HSD1 inhibitor could improve glycometabolic control and reduce the fracture risk at the same time.

At present, however, the clinical usefulness of the cortisol “milieu” assessment for individuating T2DM patients at risk of fracture is still to be determined.

### 4.8. Hydrogen Sulfide

Hydrogen sulfide (H_2_S) is a new gaseous signaling molecule which acts as a key messenger in many physiological and pathological conditions. Endogenously, H_2_S is produced within cells by the catabolic pathway of sulfurated amino acids, also known as the transsulfuration pathway, by means of the two enzymes cystathionine beta-synthase (CBS) and cystathionine gamma-lyase (CSE) [155]. Physiologically, H_2_S freely diffuses through cell membranes and is released in the circulation, where it can be present in the form of free H_2_S or bound sulfane sulfur. Decreased nitric oxide (NO) bioavailability and deficiency of H_2_S are considered to be involved in the pathophysiology of both T2DM [155] and osteoporosis [156]. Cystathionine beta-synthase is abundantly expressed in several tissues and in particular in BMSC, in insulin-secreting pancreatic *β*-cells, and several studies showed a role of H_2_S in both inhibition of insulin secretion mediated by ATP-sensitive K+ channels and a pro- or antiapoptotic effects on *β*-cells [157] and in skeletal muscles. Most studies indicate that in both animal models of diabetes and T2DM patients, H_2_S blood levels are decreased.

In BMSC, H_2_S has a fundamental role for maintaining cell proliferation and differentiation [158]. Indeed, H_2_S deficiency in BMSC attenuates both osteogenesis and proliferation. In keeping with this, CBS-deficient mice have decreased serum and intracellular levels of H_2_S and a severe osteoporotic phenotype [158, 159]. The H_2_S administration to CBS deficient mice of can restore normal bone homeostasis [158]. One of the supposed mechanisms is the increase of Hcy that leads to and oxidative damage and dysfunction of the BMMSCs. Moreover, several studies showed that osteoporosis derived from estrogen deficiency is associated to a defective H2S biosynthesis [160] and the treatment with an H2S donor prevents the bone loss induced by stimulating bone formation through the activation of the Wnt signaling cascade by increased production of the Wnt ligands.

H2S regulated insulin sensitivity, gluconeogenesis, and glycogenolysis and inhibits glucose utilization and glycogen storage. It seems also to regulate adipose tissue lipolysis, adipokine production, and inflammation, processes important for local and systemic insulin sensitivity [161] (Figure 2).

Recently, a study showed, in a rat model of diabetes, a reduced expression of CBS and other enzymes involved in H2S production in skeletal muscles and suggested a possible relationship between sarcopenia and H2S deficiency. Indeed, in this animal model, the treatment with H2S donor showed to lead to an improvement in muscle mass and functionality (Figure 3).

Nowadays, although being an extremely promising research field, H2S cannot be considered among the drugs possibly available in the very next future.

## 5. Conclusions

Nowadays, reduced bone quality and an increased fracture risk should be considered among the possible complications of T2DM. In T2DM individuals, the risk of fractures is increased for a given BMD and bone turnover markers are relatively low in these patients. These features explain the difficulty in identifying patients at high fracture risk, since physicians could not rely on the BMD measurement and/or on bone turnover assessment.

Notwithstanding the current limitations, the increasing knowledge regarding the pathophysiology of the T2DM-related bone damage gives us some information regarding which T2DM individual may be at higher risk for bone fragility. Indeed, T2DM patients with longer (≥10 years) disease duration, insulin use, poor glycometabolic control, and diabetic complications are predisposed to fracture and, in these subjects, beside the BMD determination, looking for vertebral morphometric fracture is advisable. Therefore, in the authors' opinion, a spinal and femur BMD determination by DXA spinal and femur BMD evaluation should be done in T2DM patients in the presence of clinical fragility fractures and/or a morphometric vertebral fracture and/or with a long T2DM duration (i.e., >10 years) and/or insulin use and/or T2DM-related chronic complication(s).

In the future, evaluating the ROS and AGE levels and the degree of cortisol secretion, peripheral activation, and sensitivity could increase our ability in predicting the fracture risk in the single T2D patient. In addition, a better understanding of the mechanisms leading to bone fragility in T2DM, such as the bone marrow fat, adipokine production, and cortisol milieu could consent to both the development of drugs able to reduce the fracture risk in T2DM and individuate those antidiabetic drugs more prone to damage the skeletal tissue. In this regard, the clinical similarities between bone damage in glucocorticoid-induced osteoporosis and T2D-related bone involvement seem to find some biological confirmation. Indeed, very recent data show that in rats, dexamethasone decreases serum H2S and two key H2S-generating enzymes in the bone marrow and the H2S treatment significantly relieved the inhibitory effect of dexamethasone on bone formation [162]. Therefore, it is possible to hypothesize that the reduced H2S levels in T2DM may depend on the increased cortisol levels at least in some diabetic patients.

In general, it is conceivable that the mechanisms underlying bone fragility are different among patients with T2DM. Therefore, the identification of the main cause of bone fragility in the single patient may consent to personalize the diagnostic approach and treatment of choice in T2DM patients at risk for fracture.

## Figures and Tables

**Table 1:** 

Metformin			
Preclinical	Ref.	Effect	
	[2, 63]	↑ Bone mass and bone strength ↓ AGE accumulation ↓ ROS formation ↓ Osteoblast apoptosis	
Clinical	Ref.	Characteristics	Fracture risk
	[46]	Prospective cohort study among 1964 Rochester residents who first met glycemic criteria for diabetes in 1970-1994 (mean age, 61.7 ± 14.0 yr; 51% men)	The risk was decreased among users of biguanides (HR, 0.7; 95% CI, 0.6-0.96)
	[64]	Prospective cohort study, based on data from the Osteoporotic Fractures in Men (MrOS) study that enrolled 5,994 men (aged ≥65 years)	Metformin did not increase the risk of nonvertebral fracture
	[65]	Case-control study based on 498,617 subjects in Denmark	Decreased risk of fractures
	[66]	Population based study among 206,672 individuals	There was no association of hip fracture with cumulative exposure to metformin
			Overall: ↓ = fracture risk

Sulfonylureas			
Preclinical	Ref.	Effect	
	[2, 61, 63]	↑ Osteoblast proliferation and differentiation	
Clinical	Ref.	Characteristics	Fracture risk
	[46]	Prospective cohort study among 1964 Rochester residents who first met glycemic criteria for diabetes in 1970-1994 (mean age, 61.7 ± 14.0 yr; 51% men)	No significant influence on fracture risk was seen with sulfonylurea
	[65]	Case-control study based on 498,617 subjects in Denmark	Use of sulfonylureas was associated with a decreased risk of any fracture
	[66]	Population-based study among 206,672 individuals	There was no association of hip fracture with cumulative exposure to sulfonylureas
	[67]	Retrospective observational study on 361,210 patients with type 2 diabetes	ICD-9-CM-coded outpatient hypoglycemic events were independently associated with an increased risk of fall-related fractures
	[69]	Cross-sectional study on 838 Japanese patients with T2DM	Decreased risk of vertebral fractures in postmenopausal women (OR = 0.48, *P* = 0.018)
			Overall: ↓ = fracture risk, ↑ fall risk due to hypoglycemia

Thiazolidinediones			
Preclinical	Ref.	Effect	
	[2, 63]	↑ Osteoclastogenesis ↑ Osteocytes apoptosis	
	[70–72]	↑ Bone marrow adipogenesis ↓ Osteoblastogenesis	
Clinical	Ref.	Design	Fracture risk
	[73]	Longitudinal study on ADOPT data from 1,840 women and 2,511 men with T2DM	The increase in fractures with rosiglitazone representing hazard ratios (95% CI) of 1.81 (1.17-2.80) and 2.13 (1.30-3.51) for rosiglitazone compared with metformin and glyburide occurred in pre- and postmenopausal women, and fractures were seen predominantly in the lower and upper limbs
	[76]	Nested case-control study based on data of 32,466 T2DM from the Longitudinal Health Insurance Database 2000 (LHID2000) and the catastrophic illness patient registry (CIPR) in Taiwan	Increased risks for fracture in patients who used TDZs, especially in female patients younger than 64 years old, for whom the risk was elevated from a 1.74- to a 2.58-fold odds ratio
			Overall: ↑ fracture risk (peripheral fractures)

Incretins			
Preclinical	Ref.	Effect	
	[2, 63]	DPP-4 inhibitors ↓ Bone resorption; ↑ trabecular and cortical bone volume	
	[82, 83]	GLP1-RA ↑ Proliferation of bone marrow mesenchymal stem cells; ↓ differentiation adipocytes; ↓ sclerostin expression	
Clinical	Ref.	Design	Fracture risk
	[85]	Meta-analysis including 16 RCTs and a total of 11,206 patients to study the risk of bone fractures associated with liraglutide or exenatide, compared to placebo or other active drugs	Liraglutide treatment was associated with a significant reduced risk of incident bone fractures (MH − OR = 0.38, 95% CI 0.17-0.87); however, exenatide treatment was associated with an elevated risk of incident bone fractures (MH − OR = 2.09, 95% CI 1.03-4.21)
	[86]	Meta-analysis including 7 RCTs to assess GLP-1Ra-related fracture risk compared with other antidiabetic drugs	Use of GLP-1Ra does not modify the risk of bone fracture in T2DM compared with the use of other antidiabetic medications
	[88]	A case-control study nested within a cohort of 1,945 diabetic outpatients with a follow-up of 4.1 ± 2.3 yr	No significant association was observed between bone fractures and medications
	[89]	A retrospective analysis of real-world data that matched 4160 DPP4i ever users to never users in metformin-treated T2DM patients (mean age 61 ± 11 yr), in Germany	The use of DPP-4 inhibitors was associated with a significant decrease in the risk of developing bone fractures (all patients HR = 0.67, 95% CI 0.54-0.84; women HR = 0.72, 95% CI 0.54-0.97; men HR = 0.62, 95% CI 0.44-0.88)
	[90]	Meta-analysis based on 51 RCTs (*N* = 36,402; mean age 57 ± 5 yr), to assess fractures in T2DM, comparing DPP-4 inhibitors with either an active agent or a placebo	No association of fracture events with the use of DPP-4 inhibitor when compared with placebo (OR; 0.82, 95% CI 0.57-1.16; *P* = 0.9) or when DPP-4 inhibitor was compared against an active comparator (OR; 1.59, 95% CI 0.91-2.80, *P* = 0.9)
			Overall: ↓ fracture risk with liraglutide; =↓ fracture risk with DPP-4 inhibitors

SGLT-2 inhibitors			
Preclinical	Ref.	Effect	
	[94]	↑ Urinary calcium ↓ Serum PTH levels	
Clinical	Ref.	Design	Fracture risk
	[92]	Meta-analysis on 20 studies including 8,286 patients treated with SGLT-2 compared with placebo	Not increased fracture risk; pooled risk ratio of bone fracture in patients receiving SGLT2 inhibitors versus placebo was 0.67 (95% confidence interval, 0.42-1.07)
	[93]	Cumulative meta-analysis of 38 RCTs (10 canagliflozin, 15 dapagliflozin, and 13 empagliflozin) involving 30,384 patients	Compared with placebo, canagliflozin (OR 1.15; 95% CI 0.71-1.88), dapagliflozin (OR 0.68; 95% CI 0.37-1.25), and empagliflozin (OR 0.93; 95% CI 0.74-1.18) were not significantly associated with an increased risk of fracture
	[96]	Randomized phase 3 study on 10,194 T2DM patients to describe the effects of canagliflozin on bone fracture risk	Fracture risk was increased with canagliflozin treatment and may be mediated by falls
			Overall: = fracture rate or ↑ by canagliflozin

Insulin			
Preclinical	Ref.	Effect	
	[97–99]	↑ Bone anabolism; ↓ bone resorption ↑ BMD	
Clinical	Ref.	Design	Fracture risk
	[46]	Prospective cohort study among 1964 Rochester residents who first met glycemic criteria for diabetes in 1970-1994 (mean age, 61.7 ± 14.0 yr; 51% men)	Increased fracture risk in patients on insulin (HR, 1.3; 95% CI, 1.1–1.5)
	[64]	Prospective cohort study, based on data from the Osteoporotic Fractures in Men (MrOS) study that enrolled 5,994 men (aged ≥65 years)	The risk of nonvertebral fracture increased only among men with T2DM who were using insulin (HR 1.74, 95% CI 1.13, 2.69)
	[43]	Prospective study on 3,654 older Australians	Insulin treatment was associated with increased fracture risk (adjusted RR 5.9, 95% CI 2.6-13.5)
	[101]	Prospective cohort study based on data from 9654 women, aged >65 yr in the Study of Osteoporotic Fractures	Insulin-treated diabetics had more than double the risk of foot (multivariate adjusted RR, 2.66; 95% CI, 1.18-6.02) fractures compared with nondiabetics
			Overall: ↑ fracture risk (especially nonvertebral fracture)
